# Small-Group Student Talk Before Individual Writing in Tertiary English Writing Classrooms in China: Nature and Insights

**DOI:** 10.3389/fpsyg.2020.570565

**Published:** 2020-09-16

**Authors:** Hui Helen Li, Lawrence Jun Zhang, Judy M. Parr

**Affiliations:** ^1^School of Foreign Languages, Wuhan University of Technology, Wuhan, China; ^2^Faculty of Education and Social Work, University of Auckland, Auckland, New Zealand

**Keywords:** sociocultural theory, EFL individual writing, nature, insights, small-group student talk

## Abstract

When exploring the nature of small-group student talk in English-as-a-foreign-language (EFL) individual writing in terms of what students are talking about, previous studies have mainly linked it to students’ writing and focused more on students’ written texts than their talk. Consequently, the analyses have largely been text-oriented rather than talk-oriented and have failed to reveal a complete picture of such talk and the socially negotiated nature of the interaction. To fill up the literature gap, we designed a study to investigate the nature of prewriting small-group student talk in Chinese tertiary EFL writing classrooms. Specifically, we examined what students were talking about when engaging in argumentative writing tasks prior to individual writing. Eight hours of audio recordings of student talk from eight small groups in two classes (*N* = 48) were collected during their prewriting small-group discussions. They were analyzed and interpreted in six categories: Content talk, language talk, task-management talk, organization talk, affective talk, and phatic talk. Major findings show that small-group student talk: (1) enabled students to generate content, language, and organization for their proceeding individual writing; (2) provided them with opportunities to facilitate collaborative linguistic problem-solving and the deliberate use of the first language (L1) for requesting and clarifying information; (3) allowed them to organize the group and scaffold each other collectively to manage the ongoing process of the task; and (4) assisted them to share their emotions and maintain group harmony at a surface level but did not help generate direct positive or negative affective expressions. Pedagogical insights into L2 writing instruction are also discussed.

## Introduction

Being deeply influenced by traditional Chinese Confucianism which highly values the status and authority of teachers (Qiu, 2011), teaching and learning of English-as-a-foreign-language (EFL) have long been viewed as teacher-centered, book-based, grammar-focused, and exam-oriented in China (Chen and Zhang, 2019). Such a context has exerted great influence on both teachers and students. On the one hand, teacher talk plays a dominant role in the classroom settings even under a “learner-centered” teaching mode (Wang, 2014) and becomes overwhelmingly powerful and authoritative. On the other hand, students are inclined to be passive, shy, and reluctant to talk. They regard reticence as a way to avoid showing off one’s oral English and a respect for others (Bao, 2014). Therefore, in order not to embarrass their peers and to help them save face, Chinese tertiary EFL students tend to remain quiet in English learning tasks (Wu, 2019). Under such a circumstance, the instruction of writing, the most challenging skill for learners in the process of learning a foreign language (Zhang, 2013), tends to focus on “error correction, dictation practice, sentence-making exercises, paragraph writing, and test-driven writing practice” (Zhang, 2016, p. 209; see also Zhang and Cheng, 2020). Such a teacher-dominant pedagogy trains students to imitate, emulate, or memorize exemplars as practical solutions to real writing problems that they encounter (Teng and Zhang, 2018, 2020), leaving few opportunities for students to collaborate and engage in peer-led small-group talk activities to consider and solve practical writing problems.

The most recent decade has witnessed a gradual gaining of the importance of student talk in writing pedagogical practices and research studies, especially after China’s National English Curriculum Standards reformed its EFL curriculum by emphasizing students’ abilities involving both the proficiency in English and the capability of critical thinking (Jin and Fan, 2011). Relevant research aligning within this context mainly focused on the exploration of student talk in pairs or groups either during collaborative writing tasks when students co-authored their written texts (Li and Zhu, 2013, 2017; Chen, 2018; Niu et al., 2018; Chen and Yu, 2019; Zhang, 2019), or in peer feedback interactions for revision after writing (Xu and Kou, 2011, 2017, 2018; Xu and Cao, 2012; Yu, 2015; Yu and Lee, 2015, 2016; Xu, 2016). Other studies that have addressed planning before writing have largely targeted individual planning by probing the effects of planning time, condition, language proficiency level, or task complexity on learners’ written texts (Ellis and Yuan, 2004; Ong and Zhang, 2010, 2013; Xing, 2015; Yi and Ni, 2015; Rahimi and Zhang, 2018, 2019; Wang and Zhang, 2019), rather than on examining leaners’ talk during collaborative planning activities together with its benefits to subsequent L2 individual writing. Little evidence from such a context, to date, has explored what students talk about when they engage in small-group student talk for the planning of individual writing.

These research foci emphasize the significance of the present study. Guided by Vygotsky’s Sociocultural Theory (1978), the current study aims to investigate the nature of small-group student talk about argumentative writing tasks before students proceed to their subsequent individual writing in the Chinese tertiary EFL writing classrooms. In doing so, it intends to elaborate and understand the nature of this talk, as well as help enrich the comparatively limited research and understanding about using student talk for writing development in the Chinese tertiary EFL context. In addition, it also seeks to provide pedagogical implications for L2 instructors regarding how to better employ and structure the small-group student talk in the writing classroom for developing students’ argumentative writing skills.

## Theoretical Framework

### Sociocultural Theory

With the emphasis on the importance of incorporating collaboration and social interaction, Sociocultural Theory (Vygotsky, 1978) posits that language, serving as a mediating tool, assists learners to co-construct knowledge and solve problems through social interactions (e.g., Powell and Kalina, 2009). From the sociocultural perspective, learning and development are embedded within social events, which occur when a learner interacts with others in the collaborative environment. Donato (2004) pointed out that “Sociocultural Theory provides this conceptual framework for description and explanation of collaboration and the learning and development it simultaneously effects” (p. 295). By concentrating on the social collaboration that all students can benefit from, sociocultural theory posits that knowledge is constructed by the group, and individual constructs are transformed as a result of group interaction. For meaningful learning, learners are encouraged to share knowledge and be actively engaged in group collaboration.

By advocating that learning is a continual movement from the current intellectual level to a higher level which more closely approximates the learner’s potential, Vygotsky (1978) claimed that this movement occurs in the zone of proximal development (ZPD) as a result of social interaction, which focuses on the distance between the actual and potential developmental levels. In line with Vygotsky’s understanding, Warschauer (1997) emphasized the importance of collaborative learning and considered it as “essential for assisting each student in advancing through his or her own ZPD” (p. 471). Support within the ZPD enables learners to bridge the gap between what is known and what can be known (Ortega, 2009). Since learning occurs in the zone of what can be known, it is essential to conceptualize ZPD as something that emerges through participation in collaborative interaction activities (Kozulin, 2002). Thus, small-group student talk in the writing classroom can provide opportunities for learners to negotiate writing tasks collectively and bridge the gap between what is known and what can be known in terms of what they have personally experienced and what is specifically required for the writing tasks.

Besides ZPD, scaffolding is also an important notion under the realm of sociocultural theory that can well support and situate small-group student talk in the writing classroom. Originally referring to expert-novice or adult-child interactions, scaffolding (Wood et al., 1976) is an assisted learning process that supports learning within the ZPD or getting to the next level of understanding of each student through the assistance of more expert others, be they teachers, peers or other adults (Powell and Kalina, 2009). From this perspective, the teacher can model the learning strategy or task and then gradually shift the responsibility to the students. Also, students can support each other in learning to accomplish tasks which are likely to be more difficult when they do them alone (Antón and Dicamilla, 1999; Ohta, 2000). By provoking the thinking process and knowledge building of the learners during small-group interactions, peer scaffolding enables the integration of the cognitive and social aspects of language by allowing peers to construct meaning within the context of dialogic interaction (Zhang, 1995). Donato (1994) proposed the notion of “collective scaffolding” by which he stated that a “knowledgeable participant can create, by means of speech, supportive conditions in which the novice can participate, and extend current skills and knowledge to higher levels of competence” (p. 40). Underpinned by such a notion, language learners, disregarding their linguistic abilities, are “at the same time individually novices and collectively experts” (Donato, 1994: p. 46), which enables learners to reach a higher level of development in small-group interactions rather than they attain it by themselves. Hence, for students to develop higher levels of understanding and achieve better performance in their writing, social interactions among students in small groups are necessary in the writing classroom.

In this sense, small-group student talk used in the writing classroom settings creates opportunities for peers to help each other with their writing by talking to and acting as analytical responders. In this way, they can become familiar with using talk as both a source and a means to develop their writing skills (Dyson, 1990). When students engage in collaborative talk, they can pool their strengths and weaknesses, bridge each other’s gaps between what is known and what can be known, and co-construct a greater knowledge as a group than any of the individuals would construct on their own (Watanabe, 2008). Furthermore, meaningful interaction and collaboration with more capable peers (van Lier, 2014), peers with equal proficiency levels (Walqui, 2006), or even the less capable peers (Kibler, 2017) in the small groups may present students opportunities to engage in different types of scaffolding with one another to support themselves for learning (van Lier, 2014). All these constructs theoretically guide the present study and enable it to interpret the nature of small-group student talk used as collaborative prewriting discussions for the planning of individual writing in the Chinese tertiary EFL writing classrooms.

## Literature Review

### Nature of Small-Group Student Talk Before Individual Writing

Small-group student talk in this study refers to the meaningful interactions among students in small groups who discuss writing tasks prior to their individual writing. Nature of small-group student talk refers to what students are talking about during the collaborative prewriting discussions. A wide range of different forms fall under the umbrella term of small-group student talk. Among them, “small-group interactions” (Storch, 2005), “collaborative dialog” (Swain, 1997; Kim and McDonough, 2008; Watanabe, 2008, 2014; Fernández Dobao, 2012, 2014), “peer group talk” (Kumpulainen, 1996), and “peer group interactions” (Cohen, 1994; Mercer, 1994; Barnes and Todd, 1995) are terms that have been used interchangeably.

Collectively, much is known about the use of student talk in pairs or small groups for developing students’ L2 writing skills in terms of when it occurs (during, or after writing) and what type of authorship it entails (collaborative or individual) (Storch, 2002, 2018; Storch and Wigglesworth, 2007; Watanabe and Swain, 2007; Shehadeh, 2011; Fernández Dobao, 2012, 2014; Storch and Aldosari, 2013; Yu, 2015; Xu, 2016; Yu and Lee, 2016; Xu and Kou, 2017, 2018). For studies in L2 concerning planning before writing, they have primarily laid emphasis on individual planning by investigating the effects of planning time, condition, language proficiency level, or task complexity on learners’ written texts (Ong and Zhang, 2010, 2013; Xing, 2015; Yi and Ni, 2015; Rahimi and Zhang, 2018, 2019; Wang and Zhang, 2019) rather than on examining leaners’ talk during collaborative planning activities together with its benefits to subsequent L2 individual writing. Comparatively, less is known about using small-group student talk as prewriting discussions for collaborative planning prior to L2 individual writing, particularly when it comes to what students are talking about during their engagement in such interactions.

Among the limited studies with respect to the nature of small-group student talk before individual writing, some explored it by comparing peer-led with teacher-led talk activities before L2 individual writing. Shi (1998a) reported that peer-led group discussions elicited a greater quantity of student talk and encouraged students to explore ideas for the content of the writing task more freely using various verbs of mental processes; however, unlike teacher-led group talk that helped students conceptualize and organize their ideas, peer-led group talk lacked a clear organization of the content. In addition, the study found that students’ drafts with a greater variety of verbs were not qualitatively better, which indicated that students might find it difficult to use these verbs efficiently for idea expression during their interactions. This early study shed light on the use of small-group student talk as collaborative prewriting discussions for L2 individual writing. However, its focus on measuring the verbs relating to mental processes in both the talk and texts from the perspective of linguistic corpora constrained the possibility of probing the nature of the dynamic process of such talk through a social lens. This lens values the nature of social relationships that are developed in students’ social activities, such as examining the types and forms of student engagement in peer-led group discussions (Kumpulainen and Mutanen, 1999). Therefore, such an analysis might not give a complete picture of the nature of small-group student talk for L2 individual writing.

Some studies have investigated the nature of talk under different conditions such as the use of different languages, the employment of structured or unstructured writing tasks, etc., Pu (2010) explored small-group student talk used as collaborative prewriting discussions in four conditions using different languages (only Chinese L1 group, only English L2 group, Chinese L1 and English L2 group, and independent planning group) in the Chinese tertiary EFL context. The study revealed that, among the three discussion groups, students elicited idea-generation most frequently, followed by talk about organization and language use, off-task talk, and task-management talk. Specifically, the study reported two major findings. Firstly, the English L2 group, together with the Chinese L1 and English L2 group, showed better group organization than the only Chinese L1 group, because each of these two groups had a group leader who volunteered to manage the ongoing negotiation of the writing task. Secondly, the English L2 group concentrated more on the task and thus elicited on-task talk most and off-task talk least, which helped explain why students in this group produced better written texts with statistically higher mean scores. On the contrary, although the other two groups generated more speech units, most of them were only repeated ones, which explained why students in these two groups failed to produce more task-related ideas, both in their talk and written texts. The study concluded that the use of English L2 in collaborative prewriting discussions enabled students to produce written texts of better quality. However, since this study merged the talk about organization and language use into one category and classified social talk into off-task talk, thus it was impossible to examine the differences between organization talk and language talk and explore the engagement through social talk. McDonough and Neumann (2014) examined small-group student talk under three conditions (i.e., unstructured prewriting discussions, structured prewriting tasks, and collaboratively-oriented students and structured prewriting tasks) by encouraging students to engage in critical reflection while brainstorming the content and organization for their paragraph writing tasks. The results showed that providing students with structured tasks may be more effective than giving general instructions, particularly for eliciting talk about organization. However, the structured prewriting tasks administered to the discussion groups of collaboration-oriented students elicited only slightly more reflective content talk than those in regular groups. Therefore, the results of the study suggested that, in order for prewriting tasks to assist students produce content and organize their ideas, it might be helpful to make explicit to students the goal of the task (both orally and on task materials) with instructions about content and organization in separate sections of the writing tasks and allocating a certain time for individual brainstorming before peer collaboration. This study mainly focused on content and organization that students elicited during their small-group discussion; other aspects, such as talk about language use, talk about task management, talk concerning emotional expressions and so on, were not covered. Thus, further investigations of these aspects are needed to understand more clearly the nature of small-group student talk.

Other studies have examined small-group student talk by emphasizing the relationship between such talk and L2 individual writing. Neumann and McDonough (2014, 2015) analyzed student talk during collaborative prewriting discussions for L2 paragraph-writing tasks, together with the relationship between student talk about content and organization and analytic ratings of L2 individual writing. The two studies revealed that the prewriting task instructions with a section about discussing organization and giving feedback to peers helped enable students to elicit more talk about organization and produce more evaluative comments. Further, texts produced following collaborative prewriting tasks were scored higher in terms of content than texts produced following the individual tasks. The studies found a moderately positive correlation between students’ reflective engagement with their peers’ ideas during the collaborative prewriting discussions and analytic ratings of the paragraphs. McDonough et al. (2018) investigated whether there were any differences in the text features and analytic ratings of paragraphs under three conditions: collaborative writing, collaborative prewriting, or no collaboration. The study identified five types of student talk, including content, organization, language, task-management, and off-task talk. The findings revealed that students rarely discussed organizational features of their paragraphs before beginning to write. Students in collaborative writing groups spent the majority of their exchanges discussing content, but the proportion of content, organization, and language episodes was significantly higher for the collaborative prewriting group. In addition, the analysis of linguistic measures indicated that the collaborative texts were more accurate than the collaborative prewriting and no collaboration texts, while the collaborative prewriting and no collaboration texts contained more subordination. Collaboration during the prewriting discussions did not lead to any differences in accuracy as compared to no collaboration texts. McDonough et al. (2019) further explored whether collaborative prewriting led to higher accuracy, complexity, or analytic ratings than individual prewriting in a solution-proposing paragraph writing task. By analyzing the transcripts of the collaborative prewriting discussions in terms of the topic of student talk (content, organization, language, task management, off-task talk), the study confirmed that students talked about content most frequently, followed by task management, organization, language, and off-task talk. Moreover, collaborative prewriting texts were more accurate and received higher ratings than the individual prewriting texts. Because these studies mainly aimed to identify the relationship between small-group student talk and the subsequent individual written texts, their analyses of such talk were largely text-oriented rather than talk-oriented and did not consider the social nature of small-group student talk.

In summary, although limited studies probed the nature of small-group student talk before L2 individual writing by analyzing the amount or type of talk, they primarily concentrated on either the comparison of students’ texts written after such talk and the teacher-led talk, or texts written after such talk under different conditions (e.g., the use of different languages, the employment of structured or unstructured writing tasks, etc.), or the exploration of the relationship between such talk and students’ written texts. In other words, the emphasis of these studies has been placed on students’ written texts, not on their talk. The categories of student talk these studies classified have largely been oriented to the elements of the written texts (e.g., content, language, and organization), which has failed to reveal a complete picture of such talk and to interpret it as a socially negotiated interaction. In addition, other important questions remain unanswered, including exploring the nature of such talk in other contexts (e.g., the Chinese tertiary EFL context) with more group members (e.g., six students) and different writing tasks (e.g., argumentative writing tasks). In order to gain a more in-depth understanding and elicit data concerning these areas, the present study attempted to answer the following research question: *What is the nature of small-group student talk when students discuss argumentative writing tasks before they proceed to individual writing in the Chinese tertiary EFL writing classrooms*?

## The Study

This study was approached within the pragmatic paradigm (Creswell, 2014) for three reasons. Firstly, small-group student talk arose out of the actions and consequences of Chinese tertiary EFL students’ social interactions in the writing classrooms, which well fitted the pragmatic paradigm that the investigated phenomena arise “out of actions, situations, and consequences rather than antecedent conditions” (Creswell, 2014, p. 43). Such a paradigm posits that these actions are socially situated and cannot be separated from the situations and contexts in which they occur” (Morgan, 2014, p. 26). Secondly, this study examined the nature of meaning-making student talk and intended to provide practical implications for L2 writing instruction by analyzing the shared meanings and joint interactions in students’ collaborative prewriting discussion. This undertaking is well matched with the pragmatic paradigm that values communication and shared meaning making in order to provide practical solutions to social problems and create “shared meanings and joint action” (Morgan, 2007, p. 67). Finally, the employment of both quantitative and qualitative measures for the analysis and interpretation in the current study was aligned with the pragmatic paradigm that assumes an independence of methods and measures and holds that the best method or measure is the one that is the most effective in producing the desired consequences of the inquiry (Teddlie and Tashakkori, 2009). Based on the pragmatic paradigm, a case study approach (Creswell, 2014) was adopted, as it would help the researcher generate rich and thick descriptions, which can “uncover the interaction of significant factors and characteristics of the phenomenon” (Merriam, 2009, p. 40).

In this study, each group consists of six students who were engaged in interactive and meaningful discussions about argumentative writing tasks before they proceeded to their subsequent individual writing in the Chinese tertiary EFL writing classrooms. The study focused on six students per group for the following two reasons. Firstly, Strijbos et al. (2004) classified three frequently used types of groups for collaborative interactions: Dyads (two members), small groups (three to six members), and large groups (seven or more). Based on the fact there are large classes in the Chinese tertiary EFL context, it is difficult for the teacher to manage too many dyads in the classroom. However, it is also challenging for the teacher to guarantee opportunities for each student to talk in groups of seven or more members within the limited class time. Secondly, the classroom desks and chairs in most of the Chinese universities are not movable, so groups must sit close to each other, which raises issues of noise level. Therefore, if more groups with fewer students in each group are formed, it will be difficult for students to hear each other clearly when all groups are discussing at the same time. Hence, small groups with six members were the best choice for this study.

### Participants and Research Context

The participants were 48 EFL sophomore students (35 females, 13 males) enrolled in an English language and literature major as an undergraduate degree program at a public university in central China. The reason for selecting English major students as the participants is that EFL writing, in most universities in China, is not taught in a specially designed writing course, except for English majors (Zhang, 2016). In the sampled university, only English major students are offered an *English Writing* course, which aims to enhance students’ basic knowledge in English writing, foster their writing competence in different genres, and cultivate their critical thinking ability. Students in other majors are all required to take a compulsory *College English* course which integrates listening, speaking, reading, writing, and translation (Zhang and Qin, 2018). Such a course mainly aims to develop students’ communicative competence in using English for study and work (Pei, 2015). These voluntary participants all grew up in China with Chinese as their mother tongue. Their mean age was 18.6 years (SD = 1.3), and they had studied English previously in primary and secondary schools for a mean of 10.6 years (SD = 1.2). Each of them took the national university entrance examination in China before they entered this university and were admitted by the English Department as intermediate-level language learners. All the major-related courses were taught in English at this public university.

The participants were enrolled in two parallel classes (24 students per class) of an EFL writing course required for their undergraduate degree in the first semester of their second year at university. All participants had completed the same university coursework before their enrolment in this writing course. The classroom instructor was an associate professor who obtained her Ph.D. degree in applied linguistics from an overseas university and had been teaching this writing course for 5 years. Both classes met the same instructor for two, 45-min class periods per week for a total of 32 class periods in a 16-week spring semester (including holidays but excluding exams). The instructor mainly adopted a genre approach for her teaching of this course. During the teaching, the instructor first introduced elements and features of a specific genre, then analyzed the target genre with model texts. After that, she modeled and co-constructed the texts with students. Finally, students were given homework assignments for independent writing, which were then handed in to the instructor for evaluation before the next class. Since teacher-fronted talk is dominant in Chinese tertiary EFL writing classes (Wang, 2016), none of these participants had any previous experience of collaborative prewriting discussions in groups of six members in any writing-related classes.

### Tasks and Procedures

Three argumentative writing tasks were selected for the small-group student talk, considering that argumentative writing is not only the most commonly tested writing genre in both national and international language tests for Chinese tertiary EFL students (Huang and Zhang, 2020) but also a widely-acknowledged assessment of L2 learners’ writing proficiency (Teng and Zhang, 2020). These argumentative writing tasks came from the battery of China’s National English As a Foreign Language Test—TEM-4, which is used to measure the English proficiency of Chinese university undergraduates majoring in English Language and Literature and to examine whether these students meet the required levels of English language abilities as specified in the National College English Teaching Syllabus for English Majors (NACFLT, 2004a, cited in Jin and Fan, 2011). TEM-4 consists of six parts, respectively, dictation, listening comprehension, language usage, cloze, reading comprehension, and writing. The writing part is vital because it accounts for 20% of the total score of 100. The reason for selecting argumentative tasks from the TEM-4 database is that these tasks could arouse participants’ interest and boost their enthusiasm to engage in this study because doing so could offer them opportunities to prepare for the test.

The procedure of data collection started from Week 8 by recruiting participants (see Table 1). In Week 9, participants in each class were arranged into four groups with six students in each group under a random selection by the classroom teacher. Altogether, eight small groups were formed across two classes. From Week 11 to 15, participants were given 20 min each week to engage in small-group student talk about an argumentative writing task prior to their individual writing. Given that participants had not previously carried out collaborative prewriting discussions in small groups of six students in a writing class, in order to familiarize them with the process, they were provided with two practice sessions from Week 11 to 12 with tasks chosen from the TEM-4 test battery. During each practice session, the instructor first handed out to each group a writing task which consisted only of the writing topic and a section regarding time and word limit, together with instructions requiring participants to state their personal opinions about the topic, giving corresponding supporting reasons, and presenting a conclusion. After that, participants were asked to discuss the writing task for 20 min without the help of any external resources. The instructor did not intervene in the students’ discussion. Accordingly, she only provided assistance when students particularly requested it. Excluding the two practice sessions, a total of approximately 8 h of small-group student talk (8 groups × 20 min × 3 tasks) were used for analysis.

**TABLE 1:** Overview of data collection procedures.

Week	Data collection
Week 8	Recruiting participants
Week 9	Group forming
Week 11	Practice sessions (20 min)
Week 12	Practice sessions (20 min)
Week 13	Task 1 (Whether College Students Should Hire Helpers to Clean Their Dormitories, 2010 TEM-4) (20 min)
Week 14	Task 2 (Whether English Majors Should Study Mathematics, 2014 TEM-4) (20 min)
Week 15	Task 3 (Whether Private Car Owners Should Be Taxed for Causing Environmental Pollution, 2011 TEM-4) (20 min)

### Data Analysis

The 8-h audio recording was transcribed by the first researcher and verified by one of her Chinese colleagues who held a Ph.D. degree in second language acquisition. A qualitative coding, to categorize small-group student talk, together with quantitative analyses of frequencies and percentages of each category, was conducted to examine the research question. The transcripts were divided into idea units, which are chunks of information, or “segments of discourse that coincide with a person’s focus of attention” (Gere and Abbott, 1985, p. 367), by using boundaries of intonation, pauses, and syntax. As the reflection of the speaker’s object of consciousness, an idea unit can be as brief as one word, such as “Yeah” or “OK.” Also, it can be as long as a single clause, such as “because it’s a waste of time and money.” More than one idea unit can be included in a single speaker turn.

To examine the research question concerning what students are talking about when they discuss argumentative writing tasks, each idea unit was categorized using a pre-determined scheme created by McDonough et al. (2019). Immediately repeated idea units were only coded once. In the process, the researchers also kept open minds and iteratively supplemented with additional inductively derived new categories or sub-categories reflecting the small-group student talk. Two new categories, affective talk and phatic talk, emerged from the data during such an analysis process. The categories, together with their definitions, are provided in Table 2, in which each category is illustrated with a representative example of small-group student talk. The examples were all directly drawn from students’ prewriting small-group discussions, so language errors were not removed.

**TABLE 2:** Modified coding scheme of small-group student talk (adapted from McDonough et al., 2019).

Category	Definition	Example
Content talk	Asking for or giving viewpoints, reasons, clarification, judgment, evaluation, and contradict or alternative ideas	A*: I came up with something we talk about before. I haven’t I haven’t say no to that about the point they are more professional than us. I have something to say. We don’t always clean our windows. Right? We never cleaned them, but the cleaners will clean them for us.*
		B*: You can do it yourself.*
		C*: You just stand yourself.*
		D*: But we’ve never done it.*
		E*: We never find a day to do it before sleeping.*
Language talk	Discussing, requesting information, or correcting errors on grammatical forms, lexical meaning, spelling, and individual words or phrases	A: Our home have hire cleaners to clean our home. And if you don’t want to, want your things to be moved, you can ask the cleaners not to move your stuff. They are experienced enough to meet your *requires*?
		B*: Request.*
		C*: Requirement.*
		D*: Requirements.*
Organization talk	Talking about the structure of paragraphs, ordering of ideas, and adding or cutting information	A: *The first is pollution.*
		B: *The second is what?*
		C: *Traffic jam*.
Task-management talk	Managing task roles; reading task instructions; requesting or giving task clarification; maintaining the ongoing process of discussion; and monitoring time	A: *So, let’s start our discussion*.
		B: Who support, who support the argument? I agree with it.
		C: *You should, you should, uh, describe*.
		D: *We can show our, we can show our opinions firstly*.
		E: *You first*.
Affective talk	Expressing positive emotions (e.g., praise, encouragement, support, thanks, and appreciation); showing negative emotions (e.g., anger, sadness, criticism, and disappointment); giving other emotional expressions (e.g., humor, apology, surprise, etc.)	A: *Help me, help me. I will help you*.
		B: *I will help you*. *To clean your air conditioning*. Hahaha…
Phatic talk	Greeting or welcoming each other; saying goodbye; backchanneling (e.g., signaling attention or involvement; exclamations; brief utterances showing agreement, understanding, certainty, and uncertainty)	A: I think you misunderstand what I mean. I mean cleaners for our dormitory is not necessary. I don’t mean that cleaner is not necessary.
		B*: Okay. Okay.*

In order to ensure the trustworthiness, all the small-group student talk was coded by the first researcher, and a subset of the total transcripts (8/24 transcripts or about 30%) was randomly selected and coded by the colleague mentioned above to check for coding consistency. The interrater reliability for coding each category was: content talk (*r* = 0.93), language talk (*r* = 0.95), organization talk (*r* = 0.96), task-management talk (*r* = 0.94), affective talk (*r* = 0.93), and phatic talk (*r* = 0.94). All the disagreements were resolved through discussions between the first researcher and her colleagues.

## Results and Discussion

The analysis of small-group student talk was presented in terms of number of idea units to show the relative proportion of each category of talk for all groups across three argumentative writing tasks. Altogether, six categories were identified in the transcribed data. Table 3 lists the total number as well as the minimum and maximum number of idea units for each category that occurred in the course of the three-session prewriting small-group discussions. The statistics show that content talk (*N* = 3193) dominated the students’ prewriting small-group discussions, followed by language talk (*N* = 301), task-management talk (*N* = 274), phatic talk (*N* = 127), affective talk (*N* = 110), and organization talk (*N* = 69). Content talk contributed the largest number of idea units, while organization talk produced the smallest. The high incidences of content, language, and task management talk demonstrate that the Chinese tertiary EFL students do use their small-group student talk to facilitate the assigned argumentative writing tasks, while the incidences of phatic and affective talk reflect that they also engage in social interactions that help them share their emotions and perform phatic functions of communication (Ong, 2003).

**TABLE 3:** Descriptive statistics of coded categories of small-group student talk.

	Number of groups	Sum	Minimum	Maximum	Mean	SD
Content talk	8	3193	340	496	399.13	69.37
Language talk	8	301	25	57	37.63	9.27
Organization talk	8	69	2	15	8.62	4.34
Task-management talk	8	274	14	78	34.25	21.43
Affective talk	8	110	3	30	13.75	11.32
Phatic talk	8	127	5	31	15.87	9.54

Such findings partially support Pu’s (2010) report that students elicited content talk most frequently but conflict with his claim that talk about organization and language use followed closely to talk about content. In addition, such results also oppose his finding that talk about task-management was least frequently elicited. These differences might be attributed to his different classifications of talk which merged the talk about organization and language use into one category and classified social talk into off-task talk. Therefore, he was unable to show the differences between organization talk and language talk. It was also impossible from the data reported to find out what types of social talk were elicited under such a classification. The results differed from McDonough et al. (2019), who analyzed collaborative prewriting discussions and reported that students talked about content most frequently, followed by task management, organization, language, and off-task talk. One possible reason why task management and organization talk occurred more frequently in their study may include the use of structured prewriting tasks in their study, which specifically instructed students to produce talk on content and organization and to provide feedback about their partner’s ideas. Therefore, it was more likely that students would focus on the content of the writing tasks and thus the tasks elicit more talk on content and organization. Another possible reason why language talk occurred less frequently in McDonough et al.’s study is that students in their study could use both L1 (Thai) and English for achieving a better understanding and collaboration, while students in the current study were told to use only English for discussion and collaboration.

Table 4 shows an overview of how the small-group student talk was categorized across each task and group. In total, 4074 idea units were identified across the three argumentative writing tasks with 1345 in Task 1, 1351 in Task 2, and 1378 in Task 3. The growth in the number of idea units reflects that, as students became more familiar with the procedures (Hurley, 2002) involved in prewriting small-group discussions, they focused more on the academic task at hand, as evidenced by the increasing amount of content talk, language talk, and organization talk across all three tasks. Conversely, there was a constant decline in the number of idea units for task-management talk (from 129 in Task 1 to 61 in Task 3), affective talk (from 56 in Task 1 to 18 in Task 3), and phatic talk (from 55 in Task 1 to 35 in Task 3).

**TABLE 4:** Coded categories of small-group student talk across tasks and groups by frequency.

	Content talk	Language talk	Organization talk	Task-management talk	Affective talk	Phatic talk	Total
**Task 1**							
Group 1	142	13	2	20	7	11	195
Group 2	150	9	3	32	10	8	212
Group 3	152	5	0	8	12	7	184
Group 4	143	2	2	26	13	1	187
Group 5	95	5	4	23	2	7	136
Group 6	101	5	1	6	2	11	126
Group 7	103	7	3	4	3	1	121
Group 8	150	7	1	10	7	9	184
Sum	1036	53	16	129	56	55	1345
**Task 2**							
Group 1	160	24	3	6	2	5	200
Group 2	165	15	4	32	13	8	237
Group 3	154	11	1	13	12	10	201
Group 4	120	13	2	8	5	4	152
Group 5	119	13	5	5	1	2	145
Group 6	109	13	2	8	2	5	139
Group 7	129	12	4	9	1	1	156
Group 8	99	15	2	3	0	2	121
Sum	1055	116	23	84	36	37	1351
**Task 3**							
Group 1	179	25	3	6	2	0	215
Group 2	181	23	5	14	4	10	237
Group 3	163	15	1	14	6	14	213
Group 4	90	14	2	15	1	0	122
Group 5	126	13	6	6	0	0	151
Group 6	134	14	3	1	1	6	159
Group 7	132	13	6	1	4	5	161
Group 8	97	15	4	4	0	0	120
Sum	1102	132	30	61	18	35	1378
Total	3193	301	69	274	110	127	4074

These findings may be attributable to two considerations. First, in order to complete the prewriting small-group discussions within 20 mins, students might give priority to the elicitation of content, language, and task management, for these were of greatest relevance to their follow-up individual writing. Second, as students were more comfortable with the process and focused more on content talk, language talk, and organization talk, they would naturally produce less task-management talk, affective talk, and phatic talk in the fixed 20 mins.

Further analysis of all categories of talk for each small group by percentage is presented in Table 5. It is apparent that content talk was fairly consistent across all groups, with a low of 72.83% and a high of 83.11% of the total idea units expressed. The high incidence of content talk in each small group is important as it demonstrates that students do use the small-group student talk to discuss the content for the assigned argumentative task. This also parallels the findings of the previous studies that students talk about content most frequently when they are engaged in collaborative prewriting tasks (e.g., Neumann and McDonough, 2015). A typical exchange of content talk is shown below. This excerpt is selected from Group 1 when students were discussing Task 1 concerning “Whether College Students Should Hire Helpers to Clean Their Dormitories”:

**TABLE 5:** Coded categories of small-group student talk for each group by percentage.

	Content talk	Language talk	Organization talk	Task-management talk	Affective talk	Phatic talk
Group 1	80%	9.42%	1.32%	5.29%	2%	2.64%
Group 2	72.83%	6%	1.76%	11.45%	3.96%	3.82%
Group 3	79.22%	4.22%	0.34%	5.91%	5.07%	5.24%
Group 4	75.75%	7.30%	1.29%	10.52%	4.08%	1.07%
Group 5	77.80%	8%	3.43%	7.78%	0.69%	2.06%
Group 6	80.19%	8.62%	1.40%	3.50%	1.17%	5.13%
Group 7	83.11%	7.31%	2.97%	3.20%	1.83%	1.60%
Group 8	81.22%	8.92%	1.64%	3.99%	1.64%	2.58%
Average	78.70%	7.52%	1.77%	6.45%	3%	3.02%

Ting: But it saves time.
Ran: Yes, I think it’s a waste of money, too. Because university students don’t have the access to earn money on their own. And they spend their parents’ money to enjoy their life. I think it’s not a good phenomenon.
Tian: And if you do cleaning and the laundry every day, you won’t waste much time.
Ting: But actually, we don’t do laundry every day.
Shi: Because we don’t have to do it every day. The space of our university dorm is not that big. So, emm, we don’t need to hire someone to clean the dormitory for us.
Yue: Emm, I think, doing the cleaning won’t waste too much time. I think maybe it’s the excuse of indulgence.
You may use the time to play computer games or do other things.

The exchanges cited above typically occurred with content talk, which mainly included asking for or giving viewpoints, reasons, clarification, judgment, evaluation, and contradicting existing ideas or providing alternative ideas. After sharing viewpoints on the topic, students argued with one another to defend and justify their viewpoints. In the process of doing so, they requested and provided reasons and clarifications. These arguments, reasons, and clarifications that students articulated were aligned with the content talk as they were “ideational aspects” (Lockhart and Ng, 1995: p. 644) of content. The most frequently occurring category of talk, exploration of ideational aspects of content, indicated that students were presented with a number of ideas from which to choose and thus could then decide which ones to incorporate into their individual written texts. This finding supported Shi’s (1998b) and Wigglesworth and Storch (2009) study that peer group talk was efficient in generating and negotiating what to write, because such a talk “provided an environment where students appeared relaxed to produce what they thought” (Shi, 1998b: p. 67).

However, when students’ ideas occurred seemingly at random during their discussions, they seldom spent time evaluating these ideas and talking about how to organize them. One possible explanation was that the argumentative writing tasks did not specifically require students to evaluate each other’s ideas and organize them into writing plans. Hence, students naturally were inclined to focus on what to write, namely, the content generation. This indicates that the naturally occurring peer-led small-group student talk alone is not enough to help students with their learning in the Chinese tertiary EFL writing classrooms. Therefore, L2 writing instructors’ scaffolding and involvement are needed to structure small-group student talk more optimally in order to promote students’ interactions about how to write. Such scaffolds might include those to help them organize ideas into writing plans. Structured argumentative writing tasks may help L2 writing instructors exploit the benefits of small-group student talk while maintaining a focus on individual writing development (McDonough and Neumann, 2014). When designing collaborative prewriting discussion activities, L2 writing instructors should place greater emphasis on evaluation and organization so that students are encouraged to challenge each other to justify ideas and transition from the discussion of content to the organization of ideas in preparation for writing.

Language talk represented an average of 7.52% of the total talk across all groups, which accounted for the second-largest proportion across all categories. In seven of the eight groups, the language talk proportion was fairly consistent, ranging from 6 to 9.42%. Only one group had a comparatively low percentage of language talk, 4.22%, which demonstrates that as students engage in collaborative talk, they are able to share their ideas and pool their knowledge to solve language-related problems. For example, when talking about Task 2, “Whether English Majors Should Study Mathematics,” students in Group 4 collaborated to facilitate linguistic problem-solving:

Yi: Tao Gong Shi (*Apply formula mechanically*)?
Qiao: Use the formula mechanically.
Yi: Use the formula mechanically to, um…
Qiao: Complete our homework. Complete our homework.
Xu: How to say Zhuan Ye Ke (*Major-related courses*)?
Qiao: Major, major courses.
Yi: Major subjects.
Xu: My point of view is based on that the critical thinking means that as the level we learned at college, it is not enough to, to build our critical thinking ability. So, how to say Bi Shang Bu Zu, Bi Xia You Yu (*worse off than some, better off than many*)? Something, something means that…
Qiao: Better than many?
Xu: Yeah, yeah, maybe.

In the above excerpt, there are three questions (excluding the repeated question) regarding lexical expressions which focus on the meaning of words. Except for the last question about a Chinese idiom that is difficult for the students, the other two are correctly resolved by group members. Such findings suggest that when engaging in small-group student talk, Chinese tertiary EFL students could bridge the gap between what each student already knew and what they could know and so collectively scaffolded each other to facilitate linguistic problem-solving. In this sense, peer group interaction is beneficial, because it presents learners with the opportunity to obtain comprehensible input that is uniquely modified for learners’ individual circumstances (Zhang, 2010; Zhang and Cheng, 2020). Pedagogically, small-group student talk, as a socially negotiated interaction, should be used to complement teacher-fronted talk so that teachers can better facilitate students’ learning in the writing classroom. If possible, teachers should provide evidence or examples to make small-group student talk a useful collaborative method for developing student’s English writing. By combining the use of small-group student talk and teacher-fronted talk, L2 instructors will be able to formulate a more effective approach to teaching writing.

In addition, although students were fully aware that they were supposed to use English in their small-group discussions, the analysis of language talk also revealed occasional use of L1 for requesting and clarifying information, particularly, the meaning of vocabulary. Such a finding corroborated the ideas of Storch and Wigglesworth (2003) that the deliberate use of L1 can contribute to a range of functions in L2 learning. It also supported Antón and Dicamilla’s (1999) claims that, within the sociocultural tradition, L2 learners, especially those with the same L1 background, use L1 as an important cognitive tool to understand the meaning of L2 and resolve L2 learning problems. L1 use has been found to exert a significant effect on L2 writing (Shi, 1998a; Zhang, 2013), and can serve important functions, such as word choice in the L2 (McDonough et al., 2016). Moreover, the use of L1 in collaborative interaction emerges as a means to create a social space in which learners are able to provide each other and themselves with help throughout the task (Antón and Dicamilla, 1999). This may enable learners to produce texts of higher complexity through employing various linguistic devices to compress more information into clauses and may also have the potential to lead to higher linguistic accuracy in the co-constructed essays (Zhang, 2018). Therefore, L2 writing instructors should allow students to use a certain amount of L1 in EFL writing classroom activities for better understanding and collaboration.

Organization talk, as shown in Table 5, only represented an average of 1.77% of the total talk across the groups, the smallest proportion of all categories of talk. The percentage for each group indicates that six out of eight groups elicited organization talk below the average percentage, which reveals that most of the groups did not talk much about the organization of ideas and structure of paragraphs. The following excerpt is selected as an example for organization talk from Group 2 when students were engaged in discussions about Task 3, Whether Private Car Owners Should Be Taxed for Causing Environmental Pollution.

Jie: This is the first point. You said two point or one point?
Song: Two points. And also…
Heng: Increase income.
Fan: You can’t mention that in an article.
Song: Um.
Fan: I think it’s easy. Your opinion is easier than us.
Chi: But it’s hard to, to…
Fan: Describe?

Here students were summarizing the points they listed as reasons for taxing private cars due to their environmental pollution. However, they failed to discuss how to arrange these points. Instead, they only checked the total number of points and evaluated whether it was easy or difficult to describe them in their writing, which indicated that students lacked a clear organization of the content they generated in discussions (Shi, 1998). Unlike Watanabe’s (2014) research reporting that, since Japanese university English learners were newly introduced to academic essay writing, they attended more to the organization rather than the content whether writing in pairs or alone, the current finding revealed that students frequently discussed content but seldom articulated how to order the generated ideas, where to place them, and how to make the links between or among ideas. This can be explained that “as the most natural way to talk about the assigned tasks” (Neumann and McDonough, 2015, p. 89); students may just prefer to “exchange ideas about content rather than create a writing plan or outlines for the ideas they were discussing” (p. 90).

Task-management talk represented an average of 6.45% of the total talk across all groups, which was the third-largest proportion across all categories, ranging from 3.20 to 11.45%. A typical exchange of task-management talk is shown below. This excerpt is selected from Group 3 when students were discussing Task 1 concerning “Whether College Students Should Hire Helpers to Clean Their Dormitories”:

Li: So now it’s your turn. There’s only one minute left.
Qin: Cleaning and doing laundry is sort of an essential skill, but not for, not in college that you should be trained, you should be trained in the primary or secondary education period.
Li: Yeah, yeah.
  ......
Qin: Okay. That’s already enough.

In this excerpt, Li, who was the group leader during most of the time in their discussions, assigned turns for group members and monitored time arrangement of the task. However, Qin, who took the leader role alternatively, finished their discussion by indicating what they said was enough. This result supported Hurley’s (2002) study that students shared the responsibility for organizing the group and managing the ongoing process of the task, rather than having someone direct the whole procedure. It can be explained by the notion that six students in a group provides more opportunities for students to assume different roles compared with groups with two to four.

Affective talk represented an average of 3% of the total talk across the groups, ranging from 0.69 to 5.07%. The following excerpt is selected as an example for affective talk from Group 3 when students were talking about Task 2, “Whether English Majors Should Study Mathematics.”

Li: So, can you tell me just how much marks you get in math?
Qin: You mean the score? 69.
Li: Sixty-nine?
Qin: Yeah.
Li: You are weaker.
Qin: I don’t think so.
Li: Yeah, you are a social science.
Qin: The exam is so easy.
Li: You are a social science. You are too weak.
Qin: I’m just not serious. Studying math build us logic thinking and ration and made us precise.

The above exchanges happened when Li pointed out that the college mathematics course for English major students was more difficult than the one in senior high school. However, Qin, who only got a score of 69 for the college mathematics course, disagreed with him by boasting that the exam was quite easy. Li then teased at Qin by pointing out that Qin was weak in mathematics because he was oriented toward the social sciences (not natural science). Finally, Qin admitted that he was just joking.

The analysis of such affective talk across all the groups found that students did express emotions like humor, which revealed the social perspective of small-group student interactions and indicated the significance of students’ social relationships. However, they did not generate direct positive and negative affective expressions. An explanation for this might be the random grouping by the classroom teacher who did not consider students’ friendships with each other. Being deeply influenced by Confucianism which values the Face Theory (King and Aono, 2017), Chinese students who do not know each other well tend to talk in a way that avoids praising themselves and others too much, bringing shame to one other, or pointing out each other’s mistakes in order to minimize potential discomfort for all members and thus maintain group harmony at a surface level (Xu and Cao, 2012). Although such superficial group harmony does reflect students’ mutual respect and politeness to each other, it may prevent students from communicating with each other more actively and freely during their interactions. Therefore, when grouping students for discussions, Chinese L2 writing instructors need to consider students’ personal relationships.

Pedagogically, Chinese L2 writing instructors need to consider the influence of traditional Chinese Confucianism with respect to students’ personal relationships and enrich their understanding of the developing writer, who is both a writer and a culturally-situated learner (Parr and Wilkinson, 2016). Moreover, since the notion of ZPD must be appreciated as an enactment that goes hand in hand with the affective domain, it cannot be fully realized and actualized by inactive participants (Sakamoto, 2017). Therefore, good social relationships may encourage students to communicate more actively and freely with each other and thus provide better opportunities for students to achieve their ZPDs. In this regard, a self-selection grouping method in the Chinese writing classrooms, that reflects students’ willingness and friendships in the composition of the groups, should be considered when grouping students for discussions. Studies supporting this method indicated that it enabled participants to be more collaborative about group work, more interested in their group members, more enthusiastic in group communication and have a higher sense of goal commitment and group accomplishment (Chapman et al., 2006).

Phatic talk only represented an average of 3.02% of the total talk across the groups, ranging from 1.07 to 5.24%. The percentage for each group indicates that five out of eight groups elicited phatic talk below the average percentage, which reveals that most of the groups did not produce much phatic talk. A typical exchange of phatic talk is shown below. This excerpt is selected from Group 6 when students were talking about Task 2, “Whether English Majors Should Study Mathematics.”

Shuang: Mathematical, mathematical questions. Maybe, maybe some basic knowledge about math is needed. And if you grasp more skills, you will get more offers.
Yu: Um, yeah.
Shuang: And if you learn Math well, when you buy vegetables or eating food, you won’t get it wrong.

The above excerpt indicated that the major exchanges happened between Shuang and Yu. After Shuang stated his reasons why he thought English major students should study Mathematics, another student, Yu, responded with “Um, yeah” to signify that he agreed with Shuang. Yu did not intend to take over the turn nor elicit a verbal response. Thus, Shuang continued talking without responding to Yu’s interruption. This was a typical backchanneling which did not indicate a turn–grabbing move but showed an understanding and agreement in terms of listenership. The signals the engaged group member sends back by using backchanneling are typically the same as those conveyed in longer stretches of phatic talk (McCarthy, 2003). Such a finding indicated that students may scaffold clarification and elaboration of each other’s utterances using phatic talk such as affirming backchanneling.

## Conclusion

As the basis for developing content for writing, talk is generative and supportive for the development as well as the articulation of ideas for writing prior to the act of transforming the ideas into written text (Parr et al., 2009). Aligning with the Sociocultural Theory perspective, the current study investigated the nature of small-group student talk used during collaborative prewriting discussions in Chinese tertiary EFL writing classrooms.

The study identified six categories of small-group student talk (content, language, organization, task-management, affective talk, and phatic talk) and yielded four major findings that small-group student talk: (a) Provided students with opportunities to generate content, language, and organization talk for their individual writing; (b) enabled them collectively to scaffold each other and pool their linguistic resources to facilitate linguistic problem-solving; (c) allowed them to share the responsibility for organizing the group and managing the ongoing process of the task; and (d) helped them share their emotions and maintain group harmony at a surface level, but they did not generate direct positive or negative affective expressions for the sake of saving others’ face and avoiding humiliation.

Although the findings of the current study may help inform L2 writing instructors’ decisions about the design and implementation of collaborative prewriting tasks in their writing classes, certain limitations should be considered for future research. Firstly, this study examined small-group student talk about argumentative writing tasks only among 48 Chinese English-major undergraduates (35 females and 13 males) and did not consider gender differences. This may pose an issue of generalizability. Furthermore, by taking each group as the analysis unit, this study did not analyze each student’s talk. Therefore, each member’s contribution to the group across all categories of talk was not identified. More studies are needed to understand talk in relation to other types of genres with analysis of both the individual and group engagement in a larger study that involves more participants. In addition, future research needs to investigate what factors may influence the nature of small-group student talk and whether there exist any longitudinal effects of small-group student talk on students’ subsequent individual writing. Finally, the exploration of L2 students’ perceptions toward the use of such talk in the writing classroom is also necessary so that L2 writing instructors can understand students’ attitudes and expectations about such talk and thus better design their classroom instructional activities.

## Data Availability Statement

All datasets presented in this study are included in the article/supplementary material.

## Ethics Statement

The studies involving human participants were reviewed and approved by University of Auckland Human Participants Ethics Committee (UAHPEC). The participants provided their written informed consent to participate in this study.

## Author Contributions

HL conceived of the initial idea, fine-tuned by LZ and JP. HL designed the study, collected and analyzed the data, and drafted the manuscript. LZ and JP revised and proofread the manuscript. All authors contributed to the article and approved the submitted version.

## Conflict of Interest

The authors declare that the research was conducted in the absence of any commercial or financial relationships that could be construed as a potential conflict of interest.
